# Efficacy of laparoscopic versus open surgery in malnourished patients with colorectal cancer

**DOI:** 10.3389/fnut.2026.1846888

**Published:** 2026-05-18

**Authors:** Qian-Tong Dong, Yi-Xin Zhang, Ruo Ling, Yan-Yun Lin, Pei-Chen Zhang, Ji Lin, Chang-Yuan Hu, Zhen Yu, Wei-Zhe Chen

**Affiliations:** 1 Department of Gastrointestinal Surgery, The First Affiliated Hospital of Wenzhou Medical University, Wenzhou, China; 2 First School of Clinical Medicine, Wenzhou Medical University, Wenzhou, China; 3 Wenzhou Medical University-Monash BDI Alliance in Clinical and Experimental Biomedicine, Wenzhou Medical University, Wenzhou, China; 4 Zhejiang Key Laboratory of Intelligent Cancer Biomarker Discovery and Translation, The First Affiliated Hospital of Wenzhou Medical University, Wenzhou, China; 5 Cancer Program, Department of Biochemistry and Molecular Biology, Biomedicine Discovery Institute, Monash University, Clayton, VIC, Australia; 6 Department of Gastrointestinal Surgery, Shanghai Tenth People's Hospital, Tongji University School of Medicine, Shanghai, China

**Keywords:** colorectal cancer, complications, laparoscopic-assisted resection, malnutrition, survival

## Abstract

**Background:**

Patients with colorectal cancer often present with malnutrition before surgery, which can negatively impact surgical outcomes. While laparoscopic surgery has demonstrated better short-term outcomes than open surgery, the potential differences in the efficacies of these two surgical approaches in malnourished patients with colorectal cancer remain unclear.

**Methods:**

This study enrolled colorectal cancer patients with Global Leadership Initiative on Malnutrition (GLIM)-defined malnutrition who underwent elective radical resection between 2015 and 2022. Patients were divided into two groups based on the surgery type. Propensity score matching (PSM) was used to balance clinicopathological characteristics between the two groups. Postoperative outcomes and survival were compared between the groups, and multivariate analysis was performed to identify independent risk factors for complications, overall survival (OS), and disease-free survival (DFS).

**Results:**

After PSM of 424 enrolled patients with malnutrition, the laparoscopic and open surgery groups each included 173 patients. Compared with the open radical resection group, the laparoscopic radical resection group exhibited lower incidences of total (*p* = 0.011) and surgical (*p* = 0.034) complications as well as a reduced postoperative hospital stay (*p* < 0.001). Laparoscopic resection was also associated with superior OS (*p* = 0.0232) and DFS (*p* = 0.0401). Finally, laparoscopic surgery was identified as an independent protective factor against total complications, surgical complications, and OS.

**Conclusion:**

Colorectal cancer patients with malnutrition who underwent laparoscopic surgery showed a lower incidence of total postoperative and surgical complications, reduced postoperative hospital stay, and improved OS and DFS. Therefore, laparoscopic surgery was associated with favorable postoperative outcomes in this specific patient population.

## 1 Introduction

Colorectal cancer is a globally prevalent malignant tumor, with an annual increase in incidence in recent years (1, 2). Surgical treatment plays a crucial role in the comprehensive management of colorectal cancer, as it is the most effective curative approach. However, patients with colorectal cancer often present with malnutrition before surgery (3, 4). Malnutrition refers to a pathological state in which the body has an insufficient intake or poor utilization of nutrients, leading to impaired bodily functions. Owing to factors such as the metabolic demands of the tumor, decreased appetite, and impaired digestive and absorptive functions, patients with colorectal cancer often undergo excessive nutrient utilization and depletion, rendering them more prone to malnutrition. Furthermore, the anatomical structure and physiological functions of the gastrointestinal tract predispose these patients to develop malnutrition or remain at the risk of it. Malnutrition negatively affects surgical outcomes in patients with colorectal cancer (5) and may also increase surgical risk, prolong postoperative recovery, and increase the incidence of surgical complications. This may be partly attributed to decreased bodily function caused by malnutrition, including impaired immune function, which renders patients more susceptible to infections, thereby further affecting surgical efficacy and patient survival (6, 7). Malnutrition is a strong predictor of morbidity, mortality, prolonged hospitalization, and readmission (8). Preoperative malnutrition in patients with colorectal cancer is associated with adverse postoperative outcomes, including postoperative weight loss, septic shock, and postoperative infections and inflammatory responses. Additionally, micronutrient deficiencies may lead to increased inflammation, decreased serum albumin levels, and elevated incidence of anastomotic leakage (9, 10). Consequently, assessing patients’ nutritional status has received significant attention from surgeons, and improving the short- and long-term prognosis of colorectal cancer patients with preoperative malnutrition is a pressing issue for clinicians.

Laparoscopic assisted resection (LAR) and open resection (OR) are the two primary surgical methods commonly used for the treatment of colorectal cancer. Compared with traditional open surgery, the advantages of laparoscopic surgery include reduced invasiveness, faster recovery, and fewer postoperative complications; hence, this method is widely used in clinical practice (11, 12). Moreover, LAR can reduce patient surgical and nursing costs and improve the efficiency of medical resource utilization (13). Several randomized controlled trials have demonstrated that laparoscopic surgery provides better short-term outcomes compared with open surgery, including less blood loss, early recovery of bowel function, fewer postoperative complications, and shorter hospital stays (14, 15). However, the potential differences in the efficacies of these two surgical approaches in malnourished patients with colorectal cancer remain inconclusive.

Therefore, this study compared the efficacy of LAR and OR in malnourished patients with colorectal cancer. We focused on the effect of malnutrition on surgical outcomes, including a comparative analysis of surgical risks, recovery times, complication rates, and long-term outcomes. Additionally, we evaluated the advantages and limitations of LAR and OR in this specific patient population to provide a more scientific basis for clinical treatment and improve the therapeutic efficacy and quality of life of patients with colorectal cancer.

## 2 Methods

### 2.1 Patients’ information

This retrospective study analyzed the clinical data of patients who underwent elective radical resection for colorectal cancer at the Department of Gastrointestinal Surgery, First Affiliated Hospital of Wenzhou Medical University, between February 2015 and February 2022. The inclusion criteria were: (1) adult patients with colorectal cancer aged ≥18 years; (2) American Society of Anesthesiologists (ASA) physical status classification ≤III; and (3) preoperative diagnosis of malnutrition based on the Global Leadership Initiative on Malnutrition (GLIM) criteria (16). The exclusion criteria were patients: (1) who underwent emergency or palliative surgery; (2) with malignant tumors at multiple sites; and (3) with missing clinical data. The data in this retrospective study were collected through an observational study registered in the China Clinical Trial Registry (No. ChiCTR2200057818) and was approved by the Medical Ethics Committee of the First Affiliated Hospital of Wenzhou Medical University. All participating patients written informed consent upon admission. This article follows the Strengthening the Reporting of Observational Studies in Epidemiology (STROBE) statement.

### 2.2 Clinical data

Patient clinical data included: age, sex, body mass index (BMI), serum albumin concentration, hemoglobin concentration, smoking and drinking history, Nutritional Risk Screening 2002 (NRS 2002) score, ASA grade, Charlson Comorbidity Index score, history of preoperative abdominal surgery, tumor-node-metastasis (TNM) stage, tumor location, tumor differentiation type, third lumbar vertebra skeletal muscle index (L3-SMI); surgical method (laparoscopy or open), operation duration, combined organ resection, stoma creation, number of lymph nodes harvested; postoperative complications (graded according to the Clavien-Dindo classification) (17), among which only grade ≥II complications were included in the present study. Grade ≥III complications were defined as severe complications. Data on postoperative hospital stay, hospitalization cost, and unplanned readmission within 30 days after discharge were also collected. Postoperative complications included surgical and medical complications. Surgical complications comprised intestinal obstruction, incisional infection, hemorrhage, peritoneal effusion, anastomotic leakage, and lymphatic leakage, whereas the medical complications included pulmonary, cardiac, venous thrombosis, persistent hypoalbuminemia, and urinary system complications. Hypoalbuminemia was defined as a serum albumin concentration <35 g/L. Anemia was defined as a hemoglobin concentration of <120 g/L in males and <110 g/L in females.

### 2.3 GLIM diagnosis of malnutrition

Patients were diagnosed according to the GLIM criteria (16), as follows: First, the patients underwent NRS 2002 nutritional risk screening, with those scoring ≥3 subjected to further evaluation. The diagnosis of GLIM-defined malnutrition required at least one phenotypic criterion (involuntary weight loss, low BMI, or reduced muscle mass) and one etiological criterion (reduced food intake or absorption, disease burden, or inflammation). Involuntary weight loss was defined as a weight loss of >5% in the past 6 months or >10% over >6 months. Reduced muscle mass was defined as a low SMI of <34.9 cm^2^/m^2^ in females and <40.8 cm^2^/m^2^ in males (18). BMI values <20 kg/m^2^ for patients aged <70 years and <18.52 kg/m^2^ for those aged ≥70 years were considered low. Because cancer is included in the disease-burden component of the etiologic criteria in the GLIM standards (19), patients in this study were classified as malnourished if they met any phenotypic criterion.

### 2.4 Preoperative nutritional management

At our center, all colorectal cancer patients scheduled for surgery undergo preliminary screening using the NRS 2002 tool upon admission. For patients identified as at nutritional risk (NRS 2002 score ≥3), routine preoperative nutritional support therapy is provided. The majority of these patients receive oral nutritional supplementation, while a smaller proportion undergo partial parenteral nutrition combined with partial enteral nutrition. In this study, all patients had an NRS-2002 score ≥3, and therefore, they all received perioperative nutritional support therapy. In addition, patients were advised to perform gentle exercises, such as walking and practicing deep breathing, prior to surgery.

### 2.5 Propensity score matching (PSM)

To minimize bias due to the nonrandom allocation among patients, we performed PSM for patients in the two groups using a multiple logistic regression model for clinicopathological covariates. First, individual propensity scores were calculated for each patient. Then patients in the two groups were paired in a 1:1 ratio using a nearest-neighbor matching algorithm without replacement, with a caliper width set to 0.2 times the standard deviation of the logit of the propensity score. Standardized mean differences (SMD) was used for PSM balance diagnosis. After PSM, we performed a statistical analysis of postoperative outcomes.

### 2.6 Follow-up

The post-discharge follow-up was mainly conducted through outpatient visits and telephone follow-ups performed every 3 months for the first 2 years after surgery, once every 6 months starting from the third year, and once yearly starting from the fifth year. Follow-up information included physical examinations; laboratory tests; ultrasound, CT, or endoscopic examinations; and overall survival (OS) and disease-free survival (DFS). The final follow-up date was June 2024.

### 2.7 Statistical analysis

Normality was tested for the continuous data. Normally distributed data are expressed as mean ± standard deviation, and were compared between groups using independent samples t-test. Non-normally distributed data are expressed as median and interquartile range and were compared between groups using the Mann–Whitney U test. Categorical variables are expressed as counts and percentages and compared using chi-square or Fisher’s exact tests. Univariate and multivariate logistic regression analyses were used to identify independent risk factors associated with postoperative complications. Postoperative survival rates were calculated using the Kaplan–Meier method. Survival curves were compared between the two groups using the log-rank test. Cox proportional hazards model analysis was used to identify the independent risk factors for OS and DFS. Data analysis was performed using IBM SPSS Statistics for Windows, version 22.0, and MedCalc, version 15.2 (MedCalc, Ostend, Belgium). Statistical significance was defined as a two-tailed *p*-value <0.05.

## 3 Results

### 3.1 Patient clinical information

**Figure 1:**
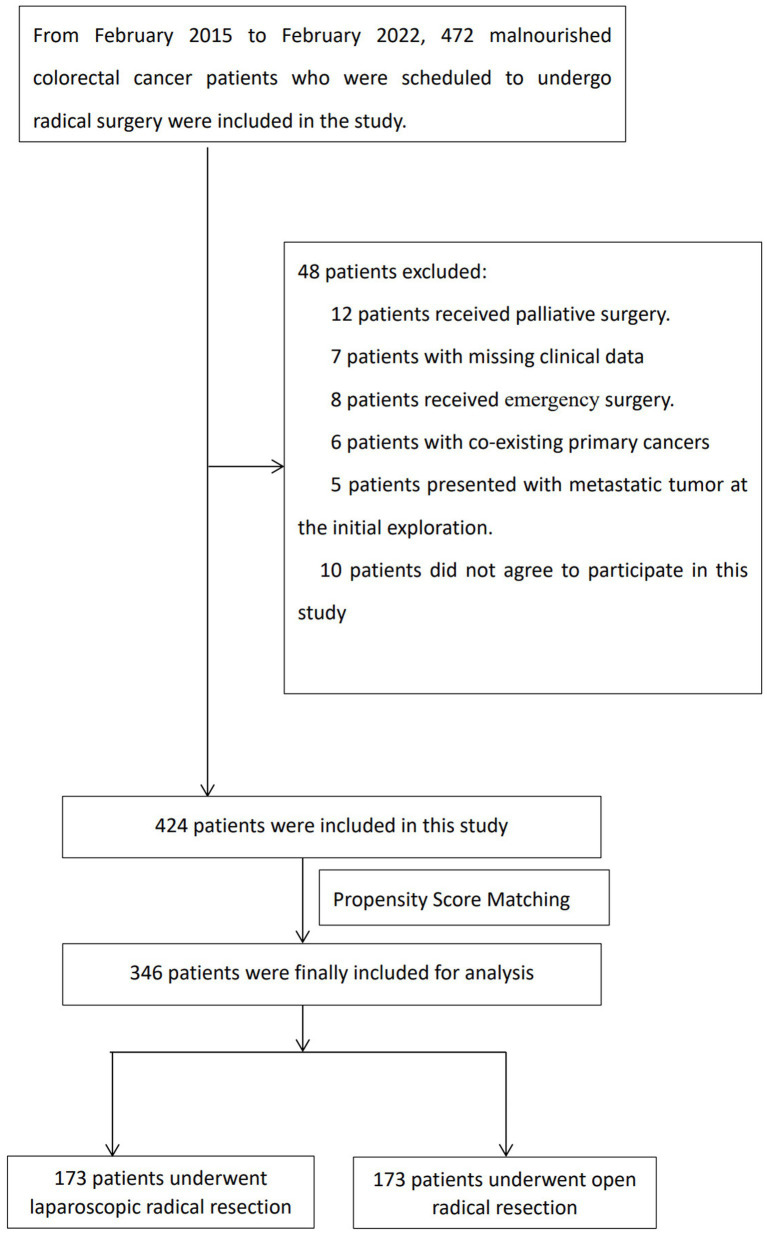
Flow chart of the present study population and group.

Table 1 shows the basic clinical information of the patients included in this study. As showed in Figure 1, among the 1,680 patients in the total database, 424 (241 males and 183 females) met the inclusion criteria and were included in the analysis, with the incidence of malnutrition being 25.2%. The median age of the patients was 70 years. The patients were divided into laparoscopic-assisted radical resection (LAR) and open radical resection (OR) groups based on surgical method. The LAR and OR groups included 216 and 208 patients, respectively. Compared with the OR group, patients in the LAR group had higher preoperative albumin levels (median, 38.0 g/L vs. 37.0 g/L, SMD = 0.20, *p* = 0.012), lower skeletal muscle index (SMI) (median, 37.0 cm^2^/m^2^ vs. 39.7 cm^2^/m^2^, SMD = 0.21, *p* = 0.002), and a lower proportion of patients with ASA grade III status (6.9% vs. 16.8%, SMD = 0.23, *p* = 0.006). Preoperative albumin levels, SMI, and ASA grade were included in the propensity model for PSM. After PSM, each group included 173 patients. The differences in the above indicators between the two groups disappeared and were comparable (SMD = 0.04, *p* = 0.865; SMD = 0.12, *p* = 0.554; and SMD < 0.01, *p* = 0.962; respectively), with relatively balanced preoperative baseline data.

**Table 1:** Demographic and clinical characteristic of patients before and after PSM.

Factors	Before PSM		SMD	*p*	After PSM		SMD	*p*
	LAR (*n* = 216)	OR (*n* = 208)			LAR (*n* = 173)	OR (*n* = 173)		
Age, median (IQR), years	70 (17)	70.5 (19)	0.04	0.401	69 (17)	70 (19)	0.09	0.262
Gender			0.07	0.459			0.01	0.914
Male	119 (55.1)	122 (58.7)			98 (56.6)	99 (57.2)		
Female	97 (44.9)	86 (41.3)			75 (43.4)	74 (42.8)		
BMI, median (IQR), kg/m^2^	21.1 (4.2)	21.2 (4.3)	0.03	0.641	21.2 (4.0)	20.9 (4.3)	0.10	0.979
Albumin, median (IQR), g/L	38.0 (6.8)	37.0 (6.8)	0.20	0.012^*^	37.7 (6.5)	37.5 (6.3)	0.04	0.865
Hemoglobin, median (IQR), g/L	115.5 (37)	115 (33)	0.02	0.919	115 (37.5)	116 (26.5)	0.04	0.563
L3 SMI, median (IQR), cm^2^/m^2^	37.0 (10.3)	39.7 (12.7)	0.21	0.002^*^	37.9 (9.7)	39.2 (12.8)	0.12	0.554
ASA grade			0.23	0.006^*^			<0.01	0.962
I	71 (32.9)	53 (25.5)			55 (31.8)	51 (29.5)		
II	130 (60.2)	120 (57.7)			103 (59.5)	113 (65.3)		
III	15 (6.9)	35 (16.8)			15 (8.7)	9 (5.2)		
NRS 2002 scores, median (IQR)	4 (1)	4 (1)	<0.01	0.476	4 (1)	4 (1)	<0.01	0.475
Charlson comorbidity index			0.13	0.116			0.04	0.704
0	106 (49.1)	90 (43.3)			84 (48.6)	82 (47.4)		
1	76 (35.2)	72 (34.6)			62 (35.8)	60 (34.7)		
≥ 2	34 (15.7)	46 (22.1)			27 (15.6)	31 (17.9)		
Smoking history	44 (20.4)	32 (15.4)	0.13	0.181	36 (20.8)	26 (15.0)	0.15	0.161
Alcohol consumption	40 (18.5)	27 (13.0)	0.15	0.118	28 (16.2)	19 (11.0)	0.15	0.158
Previous abdominal surgery	48 (22.2)	54 (26.0)	0.09	0.368	39 (22.5)	45 (26.0)	0.08	0.452
Tumor location			0.23	0.053			0.14	0.413
Right hemicolon	63 (29.2)	84 (40.4)			54 (31.2)	65 (37.6)		
Left hemicolon	77 (35.6)	63 (30.3)			61 (35.3)	52 (30.1)		
Rectum	76 (35.2)	61 (29.3)			58 (33.5)	56 (32.3)		
Differentiation of tumor			0.17	0.083			0.19	0.087
Well differentiated	176 (81.5)	155 (74.5)			143 (82.7)	130 (75.1)		
Poorly differentiated	40 (18.5)	53 (25.5)			30 (17.3)	43 (24.9)		
TNM stage			0.06	0.508			0.08	0.400
I	40 (18.5)	35 (16.8)			33 (19.1)	30 (17.4)		
II	90 (41.7)	84 (40.4)			77 (44.5)	72 (41.6)		
III	86 (39.8)	89 (42.8)			63 (36.4)	71 (41.0)		

Values in parentheses are percentages unless indicated otherwise.

^*^Statistically significant (*p* < 0.05).

PSM, propensity score match; SMD, standardized mean differences; LAR, laparoscopic-assisted radical resection; OR, open radical resection; IQR, interquartile range; BMI, body mass index; ASA, American Society of Anesthesiologists; L3 SMI, third lumbar vertebra skeletal muscle index; NRS, nutritional risk screening; TNM, tumor–node–metastasis.

### 3.2 Postoperative clinical outcomes

The results of the analysis of the short-term postoperative clinical outcomes are detailed in Table 2. After PSM, a total of 82 patients developed postoperative complications, corresponding to an incidence rate of 23.7%. Compared with the OR group, patients in the LAR group had a relatively lower incidence of postoperative (17.9% vs. 29.5%, *p* = 0.011) and surgical complications (13.3% vs. 22.0%, *p* = 0.034), with no statistically significant differences in medical complications between the two groups (6.3% vs. 8.7%, *p* = 0.415). Specifically, the incidence of incision infection (3.5% vs. 11.0%, *p* = 0.007) and intestinal obstruction (1.2% vs. 5.2%, *p* = 0.032) was significantly lower in the LAR group. Moreover, patients in the LAR group had a significantly shorter postoperative hospital stay (median, 10 days vs. 13 days, *p* < 0.001), longer operation time (median, 170 min vs. 150 min, *p* = 0.001), and higher hospitalization costs (57880.7 yuan vs. 48237.6 yuan, *p* < 0.001) compared with patients in the OR group. Unplanned readmission within 30 days after discharge did not differ significantly between the two groups (3.5% vs. 2.9%, *p* = 0.759). During hospitalization and the 30-day follow-up period after discharge, one patient in the OR group died.

**Table 2:** Details of postoperative outcomes.

Factors	Total (*n* = 346)	LAR (*n* = 173)	OR (*n* = 173)	*p*
Total complications	82 (23.7)	31 (17.9)	51 (29.5)	0.011^*^
Surgical complications	61 (17.6)	23 (13.3)	38 (22.0)	0.034^*^
Wound infection	25 (7.2)	6 (3.5)	19 (11.0)	0.007^*^
Bleeding	4 (1.2)	1 (0.6)	3 (1.7)	0.623
Intra-abdominal abscess	12 (3.5)	7 (4.0)	5 (2.9)	0.557
Anastomotic leakage	11 (3.2)	6 (3.5)	5 (2.9)	0.759
Intestinal obstruction	11 (3.2)	2 (1.2)	9 (5.2)	0.032^*^
Lymphorrhagia	2 (0.6)	2 (1.2)	0 (0)	0.499
Medical complications	26 (7.5)	11 (6.4)	15 (8.7)	0.415
Pulmonary complications	9 (2.6)	3 (1.7)	6 (3.5)	0.502
Cardiac complications	3 (0.9)	1 (0.6)	2 (1.2)	1.000
Venous thrombosis	5 (1.4)	2 (1.2)	3 (1.7)	1.000
Persistent hypoalbuminemia	6 (1.7)	2 (1.2)	4 (2.3)	0.685
Urinary infection	5 (1.4)	3 (1.7)	2 (1.2)	1.000
Severe complications	18 (5.2)	8 (4.6)	10 (5.8)	0.682
Combined organ resection	25 (7.2)	15 (8.7)	10 (5.8)	0.299
Stoma	28 (8.1)	14 (8.1)	14 (8.1)	1.000
Number of lymph node harvested, median (IQR)	17 (3)	17 (3)	17 (3)	0.816
Surgical durations, median (IQR), minutes	160 (78)	170 (80)	150 (60)	0.001^*^
Postoperative hospital stays, median (IQR), days	11.5 (6)	10 (6)	13 (7)	<0.001^*^
Costs, median (IQR), yuan	54799.0 (20,158.1)	57880.7 (18550.7)	48237.6 (21800.6)	<0.001^*^
Readmissions within 30 days of discharge	11 (3.2)	6 (3.5)	5 (2.9)	0.759

Values in parentheses are percentages unless indicated otherwise.

^*^Statistically significant (*p* < 0.05).

LAR, laparoscopic-assisted radical resection; OR, open radical resection; IQR, interquartile range.

### 3.3 Risk factor analysis of postoperative complications

Multivariate analysis showed that hypoproteinemia (OR = 1.963, 95% CI 1.155–3.337, *p* = 0.013), rectal cancer (OR = 1.775, 95% CI 1.038–3.034, *p* = 0.036), and combined organ resection (OR = 2.755, 95% CI 1.140–6.660, *p* = 0.024) were risk factors for postoperative complications in patients with colorectal cancer with malnutrition, whereas laparoscopic surgery was a protective factor (OR = 0.490, 95% CI 0.291–0.826, *p* = 0.007) (Table 3).

**Table 3:** Analysis of risk factors for total complications.

Factors	Univariate analysis	*p*	Multivariate analysis	*p*
	OR (95% CI)		OR (95% CI)	
Age				
≥65/<65	1.690 (0.977–2.923)	0.061		
Gender				
Male/female	0.738 (0.449–1.214)	0.232		
BMI				
≤ 18.5/18.5–25	1.136 (0.615–2.098)	0.684		
>25/18.5–25	1.311 (0.595–2.887)	0.502		
ASA grade				
III/I, II	1.356 (0.542–3.394)	0.515		
Hypoalbuminemia				
Yes/no	1.842 (1.104–3.075)	0.019^*^	1.963 (1.155–3.337)	0.013^*^
Anemia				
Yes/no	1.024 (0.623–1.682)	0.925		
TNM stage				
III/I	2.735 (1.068–5.283)	0.034^*^		
II/I	1.842 (0.827–4.103)	0.135		
Charlson comorbidity index				
1/0	1.220 (0.700–2.128)	0.482		
≥2/0	1.552 (0.788–3.055)	0.203		
Previous abdominal surgery				
Yes/no	0.699 (0.379–1.288)	0.251		
Differentiation of tumor				
Poorly/Well	1.668 (0.942–2.956)	0.079		
Tumor location				
Rectum/Colon	1.522 (0.911–2.542)	0.099	1.775 (1.038–3.034)	0.036^*^
Combined resection				
Yes/no	2.306 (0.993–5.351)	0.052	2.755 (1.140–6.660)	0.024^*^
Laparoscopic-assisted surgery				
Yes/no	0.522 (0.314–0.868)	0.012^*^	0.490 (0.291–0.826)	0.007^*^

^*^Statistically significant (*p* < 0.05).

OR, odds ratio; CI, confidence interval; BMI, body mass index; ASA, American Society of Anesthesiologists; TNM, tumor–node–metastasis.

Hypoproteinemia (OR = 2.480, 95% CI 1.386–4.437, *p* = 0.002) and rectal cancer (OR = 2.064, 95% CI 1.149–3.709, *p* = 0.015) were risk factors for surgical complications in malnourished patients with colorectal cancer, while laparoscopic surgery was a protective factor (OR = 0.531, 95% CI 0.297–0.948, *p* = 0.032) (Table 4).

**Table 4:** Analysis of risk factors for surgical complications.

Factors	Univariate analysis	*p*	Multivariate analysis	*p*
	OR (95% CI)		OR (95% CI)	
Age				
≥65/<65	1.533 (0.834–2.818)	0.169		
Gender				
Male/female	0.803 (0.461–1.398)	0.437		
BMI				
≤ 18.5/18.5–25	0.933 (0.462–1.886)	0.848		
>25/18.5–25	1.126 (0.463–2.743)	0.793		
ASA grade				
III/I, II	1.250 (0.448–3.489)	0.670		
Hypoalbuminemia				
Yes/no	2.244 (1.276–3.944)	0.005^*^	2.480 (1.386–4.437)	0.002^*^
Anemia				
Yes/no	0.870 (0.499–1.517)	0.624		
TNM stage				
III/I	2.113 (0.868–5.142)	0.099		
II/I	1.691 (0.693–4.128)	0.249		
Charlson comorbidity index				
1/0	1.261 (0.687–2.315)	0.455		
≥2/0	1.073 (0.484–2.378)	0.863		
Previous abdominal surgery				
Yes/no	0.915 (0.475–1.761)	0.790		
Differentiation of tumor				
Poorly/Well	1.580 (0.840–2.971)	0.156		
Tumor location				
Rectum/Colon	1.807 (1.028–3.179)	0.040^*^	2.064 (1.149–3.709)	0.015^*^
Combined resection				
Yes/no	1.527 (0.583–3.999)	0.389		
Laparoscopic-assisted surgery				
Yes/no	0.545 (0.309–0.961)	0.036^*^	0.531 (0.297–0.948)	0.032^*^

^*^Statistically significant (*p* < 0.05).

OR, odds ratio; CI, confidence interval; BMI, body mass index; ASA, American Society of Anesthesiologists; TNM, tumor–node–metastasis.

### 3.4 Analysis of long-term postoperative outcomes and risk factors

**Figure 2:**
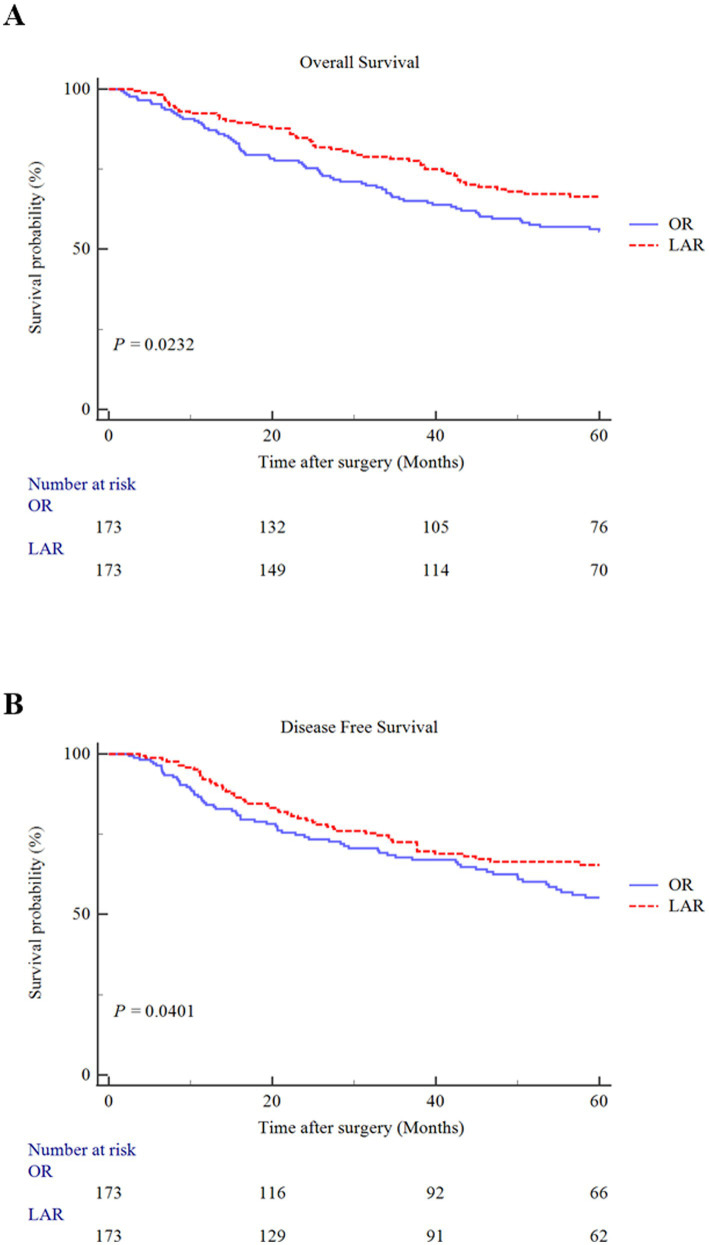
Kaplan–Meier curves for overall survival **(A)** and disease-free survival **(B)** in colorectal cancer patients with malnutrition underwent laparoscopic-assisted radical resection (LAR) or open radical resection (OR) after propensity score match.

The median postoperative follow-up time was 52 months. Figure 2 shows the OS and DFS curves of colorectal cancer patients with malnutrition after PSM. Patients in the LAR group had better OS (*p* = 0.0232) and DFS (*p* = 0.0401) after surgery compared with patients in the OR group.

Older age (HR = 1.911, 95% CI 1.306–2.795, *p* = 0.001) and TNM stage III (HR = 1.637, 95% CI 1.027–2.610, *p* = 0.038) were risk factors for postoperative OS in colorectal cancer patients with malnutrition, whereas LAR was a protective factor (HR = 0.689, 95% CI 0.492–0.965, *p* = 0.030) (Table 5).

**Table 5:** Analysis of risk factors for overall survival.

Factors	Univariate analysis	*p*	Multivariate analysis	*p*
	HR (95% CI)		HR (95% CI)	
Age				
≥65/<65	1.825 (1.249–2.666)	0.002^*^	1.911 (1.306–2.795)	0.001^*^
Gender				
Male/female	0.781 (0.560–1.089)	0.145		
BMI				
≤ 18.5/18.5–25	1.536 (1.048–2.252)	0.028^*^		
>25/18.5-25	0.821 (0.450–1.500)	0.522		
ASA grade				
III/I, II	0.918 (0.467–1.804)	0.804		
Hypoalbuminemia				
Yes/no	1.568 (1.118–2.199)	0.009^*^		
Anemia				
Yes/no	1.182 (0.848–1.646)	0.324		
TNM stage				
III/I	1.576 (0.990–2.511)	0.055	1.637 (1.027–2.610)	0.038^*^
II/I	0.844 (0.517–1.379)	0.499		
Charlson comorbidity index				
1/0	1.220 (0.843–1.765)	0.292		
≥2/0	1.341 (0.851–2.112)	0.863		
Previous abdominal surgery				
Yes/no	0.805 (0.535–1.211)	0.297		
Differentiation of tumor				
Poorly/Well	1.550 (1.060–2.266)	0.024^*^		
Tumor location				
Rectum/Colon	0.999 (0.703–1.419)	0.997		
Combined resection				
Yes/no	0.889 (0.452–1.747)	0.733		
Laparoscopic-assisted surgery				
Yes/no	0.679 (0.485–0.950)	0.024^*^	0.689 (0.492–0.965)	0.030^*^

^*^Statistically significant (*p* < 0.05).

HR, hazard ratio; CI, confidence interval; BMI, body mass index; ASA, American Society of Anesthesiologists; TNM, tumor–node–metastasis.

Additionally, older age (HR = 2.308, 95% CI 1.520–3.505, *p* < 0.001) and TNM stage III (HR = 2.035, 95% CI 1.198–3.456, *p* = 0.009) were also risk factors for postoperative DFS in colorectal cancer patients with malnutrition (Table 6).

**Table 6:** Analysis of risk factors for disease-free survival.

Factors	Univariate analysis	*p*	Multivariate analysis	*p*
	HR (95% CI)		HR (95% CI)	
Age				
≥65/<65	2.200 (1.450–3.337)	<0.001^*^	2.308 (1.520–3.505)	<0.001^*^
Gender				
Male/female	0.894 (0.626–1.276)	0.536		
BMI				
≤ 18.5/18.5–25	1.167 (0.751–1.814)	0.492		
>25/18.5–25	1.033 (0.586–1.820)	0.911		
ASA grade				
III/I, II	1.331 (0.716–2.472)	0.366		
Hypoalbuminemia				
Yes/no	1.616 (1.124–2.324)	0.010^*^		
Anemia				
Yes/no	0.899 (0.630–1.284)	0.558		
TNM stage				
III/I	1.946 (1.146–3.303)	0.014^*^	2.035 (1.198–3.456)	0.009^*^
II/I	1.136 (0.659–1.956)	0.647		
Charlson comorbidity index				
1/0	0.911 (0.604–1.374)	0.657		
≥2/0	1.516 (0.964–2.384)	0.071		
Previous abdominal surgery				
Yes/no	0.959 (0.632–1.454)	0.844		
Differentiation of tumor				
Poorly/Well	1.574 (1.047–2.367)	0.029^*^		
Tumor location				
Rectum/Colon	0.945 (0.648–1.378)	0.768		
Combined resection				
Yes/no	1.149 (0.602–2.194)	0.674		
Laparoscopic-assisted surgery				
Yes/no	0.689 (0.482–0.985)	0.041^*^		

^*^Statistically significant (*p* < 0.05).

HR, hazard ratio; CI, confidence interval; BMI, body mass index; ASA, American Society of Anesthesiologists; TNM, tumor–node–metastasis.

## 4 Discussion

This study compared the efficacies of two surgical approaches in colorectal cancer patients with GLIM malnutrition. Compared with patients who underwent OR, those who underwent LAR had lower incidences of total postoperative and surgical complications, shorter postoperative hospital stays, longer operation times, higher hospitalization costs, and better postoperative OS and DFS. The results of the multivariate analysis showed that laparoscopic surgery was a protective factor against total postoperative complications, surgical complications, and postoperative OS in malnourished patients with colorectal cancer.

To our knowledge, this is the first study to compare the prognoses of malnourished colorectal cancer patients treated with different surgical approaches. The advantages of LAR in colorectal cancer treatment include minimal invasiveness, mild postoperative pain, rapid recovery of intestinal function, and shorter hospital stay (20, 21). Nutritional status is a key factor influencing surgical prognosis in patients with colorectal cancer, and significantly increases the risk of postoperative complications, prolongs recovery time, and reduces treatment tolerance (22). Malnourished patients also have significantly higher surgical and infectious complication rates (3). We previously demonstrated that malnutrition negatively affects postoperative outcomes in patients with colorectal cancer (8).

For patients with malnutrition or nutritional risk, guidelines recommend preoperative nutritional support therapy or nutritional prehabilitation for at least 7–10 days (22, 23). However, malnutrition cannot be completely reversed in the short term. Therefore, whether surgical approach can improve the prognosis of malnourished patients undergoing surgery warrants exploration. Additionally, to eliminate nonrandom allocation bias, we used PSM to balance preoperative baseline characteristics between the two groups, enhancing the credibility of the results.

Laparoscopic radical resection of colorectal cancer, with its minimally invasive advantages, has become a core component of the Enhanced Recovery After Surgery (ERAS) concept (24, 25). This surgical approach is particularly critical in malnourished patients. Minimally invasive surgery minimizes postoperative complications, which are the primary causes of nutritional depletion and delayed recovery (26). Second, reduced postoperative pain enables earlier ambulation, effectively maintaining muscle strength and slowing muscle loss caused by bed rest and the disease itself. Most importantly, the rapid recovery of intestinal function after LAR significantly shortens the fasting time, allowing patients to receive early oral feeding or enteral nutritional support (27). This ensures that valuable proteins and energy are promptly used for tissue repair, immune function maintenance, and protein synthesis rather than being consumed to repair extensive surgical trauma and address complications. Thus, laparoscopic technology breaks the vicious cycle of “surgical consumption-inability to eat-worsening malnutrition” in malnourished patients by reducing trauma, lowering complications, and enabling early oral nutrition, directly driving the achievement of ERAS goals (28, 29).

The results of the present study showed that LAR, significantly reduced the incidence of total and surgical complications, with particular advantages in reducing incision infections and intestinal obstruction. Traditional OR requires a long abdominal incision, which leads to extensive tissue exposure that is prone to contamination, compromised blood supply, and poor healing. In contrast, LAR requires only several small trocar ports, greatly reducing abdominal wall trauma and tissue exposure, thereby fundamentally lowering the risk of bacterial invasion and infection (30). In addition, reduced intraoperative intestinal compression lowers the risk of bacterial translocation. In OR, the prolonged exposure of intra-abdominal organs to air, surgical manipulation, and inevitable tissue retraction can easily damage the peritoneum and intestinal serosa, triggering inflammation and fibrinous exudation, which leads to adhesive intestinal obstruction (31). LAR operates within a closed abdominal cavity, avoiding organ exposure and serosal damage from gauze wiping. Additionally, precise instrument manipulation also reduces unnecessary tissue contact, significantly decreasing postoperative intra-abdominal adhesions and fundamentally preventing adhesive small bowel obstruction (32, 33). Other studies have shown that LAR significantly reduces the overall incidence of postoperative intestinal obstruction compared with OR (34, 35).

Previous studies have suggested that laparoscopic surgery is non-inferior to open surgery in terms of long-term survival (36, 37), whereas the present study found that laparoscopic colorectal cancer surgery is superior to open surgery in terms of OS. Malnourished patients exhibit poor immune function, impaired tissue healing, and low tolerance to major surgery (38). The minimally invasive nature of LAR significantly reduces the overall incidence of postoperative complications (39). This allows patients to recover more quickly from surgical stress, accept and tolerate the necessary adjuvant chemotherapy earlier, complete the full comprehensive treatment regimen, and fundamentally improve their long-term survival prognosis. Thus, the survival benefit does not stem from the surgical approach itself but rather from protecting the patient’s physical condition and ensuring the smooth implementation of subsequent treatment. In contrast, DFS mainly depends on the biological behavior of the tumor and the oncological radicality of surgery (e.g., negative surgical margins and adequate lymph node dissection). Moreover, laparoscopic technology itself cannot alter tumor malignancy, and is therefore not generally considered an independent protective factor for DFS, consistent with the conclusions of the present study (40).

This study has several limitations. First, owing to the retrospective design, the study had inherent and unavoidable selection bias. Although PSM was used to balance preoperative baseline data between the laparoscopic and open surgery groups, this method could not control for unrecorded or unmeasurable confounding factors. Thus, the evidentiary strength of the conclusions was inferior to that of prospective RCTs. Future well-designed, multicenter, large-sample RCTs are urgently needed to strengthen the evidence. Second, the preoperative nutritional support protocols used in this study were heterogeneous, with no unified standards for specific implementation strategies, routes (enteral or parenteral), duration, or energy/protein intake goals. Future studies should incorporate standardized nutritional support protocols as core design components to accurately evaluate the independent effects of surgical approach. Finally, not all patients were included in the ERAS pathway management. Thus, the observed outcome differences may partially result from variations in ERAS implementation. Future research should compare the efficacies of the two surgical techniques in this specially malnourished population under the premise of a comprehensive, unified ERAS implementation to derive more robust, reliable, and clinically meaningful conclusions.

In conclusion, in this retrospective analysis, laparoscopic surgery was associated with favorable postoperative outcomes in malnourished colorectal cancer patients.

## Data Availability

The raw data supporting the conclusions of this article will be made available by the authors, without undue reservation.
